# Inflammation mediates the effect of adiposity and lipid metabolism indicators on the embryogenesis of PCOS women undergoing *in vitro* fertilization/intracytoplasmic sperm injection

**DOI:** 10.3389/fendo.2023.1198602

**Published:** 2023-07-25

**Authors:** Huahua Jiang, Lixue Chen, Tian Tian, Huifeng Shi, Ning Huang, Hongbin Chi, Rui Yang, Xiaoyu Long, Jie Qiao

**Affiliations:** ^1^ Center for Reproductive Medicine, Department of Obstetrics and Gynecology, Peking University Third Hospital, Beijing, China; ^2^ National Clinical Research Center for Obstetrics and Gynecology, Peking University Third Hospital, Beijing, China; ^3^ Key Laboratory of Assisted Reproduction, Peking University, Ministry of Education, Beijing, China; ^4^ Beijing Key Laboratory of Reproductive Endocrinology and Assisted Reproductive Technology, Beijing, China; ^5^ Department of Obstetrics and Gynecology, Peking University Third Hospital, Beijing, China; ^6^ National Centre for Healthcare Quality Management in Obstetrics, Beijing, China; ^7^ Beijing Advanced Innovation Center for Genomics, Peking University, Beijing, China; ^8^ Peking-Tsinghua Center for Life Sciences, Peking University, Beijing, China

**Keywords:** PCOS, adiposity, lipid metabolism, inflammation, mediating effect, IVF/ICSI outcomes

## Abstract

**Background:**

Polycystic ovary syndrome (PCOS) is a complex reproductive endocrine and metabolic disease affecting women of reproductive age. The low-grade chronic inflammation in PCOS is considered to be associated with obesity and dyslipidemia. We aim to investigate the potential mediating role of white blood cell (WBC) count, a representative inflammatory marker, in the effect of adiposity and lipid metabolism indicators on IVF/ICSI outcomes in PCOS women.

**Methods:**

We conducted a retrospective cohort study of 1,534 PCOS women who underwent their first IVF/ICSI cycles with autologous oocytes at a reproductive center from January 2018 to December 2020. The associations between PCOS women’s adiposity and lipid metabolism indicators and WBC count and IVF/ICSI outcomes were examined using multivariable generalized linear models. Mediation analyses were conducted to evaluate the possible mediating role of WBC count.

**Results:**

We found significant dose-dependent correlations between adiposity and lipid metabolism indicators and IVF/ICSI outcomes (i.e., hormone levels on the ovulatory triggering day, oocyte development outcomes, fertilization, early embryo development outcomes, and pregnancy outcomes) (all *p* < 0.05), as well as between adiposity and lipid metabolism indicators and WBC count (all *p* < 0.001). Increasing WBC count was associated with adverse oocyte and embryonic development outcomes (all *p* < 0.05). Mediation analyses suggested that increasing serum TG and LDL-C levels and decreasing serum HDL-C level were significantly associated with reduced high-quality Day 3 embryo count in PCOS women, with 21.51%, 9.75%, and 14.10% mediated by WBC count, respectively (all *p* < 0.05).

**Conclusions:**

We observed significant associations between lipid metabolism indicators and high-quality Day 3 embryo count in PCOS women, partially mediated by inflammation-related mechanisms, suggesting the potential intervention target for improving embryo quality in PCOS women.

## Introduction

Polycystic ovary syndrome (PCOS) is a lifelong reproductive, metabolic, and psychological condition affecting 5% to 18% of reproductive-aged women (1). The primary clinical manifestations of PCOS are oligo-anovulation, hyperandrogenism (clinical or biochemical), and polycystic ovarian morphology (2). PCOS women have high risks of anovulatory infertility, dyslipidemia, obesity, impaired glucose tolerance (IGT), insulin resistance (IR), type 2 diabetes mellitus (T2DM), cardiovascular disease, gynecological cancers, and psychiatric disorders (1, 3–5). PCOS is a heterogeneous and complex multifactorial disorder resulting from the combined influences of genetic factors, environment, endocrine, dietary habits, and lifestyle (1, 5–7). Increasing evidence has shown that PCOS is closely related to obesity and its relevant metabolic disorders (3).

Obesity, affecting approximately 15% of women worldwide, is a chronic metabolic disease and has grown to epidemic proportions over the past few decades (8). The prevalence of obesity in PCOS women ranges from 50% to 80%, which is approximately three times higher than in women without PCOS (6). The significant association between body mass index (BMI) and PCOS characteristics, regardless of age, has been elucidated by the Northern Finnish Birth Cohort (NFBC) study group as early as the 1960s (9). Obesity promotes and amplifies all endocrine, metabolic, and reproductive outcomes in PCOS women, including biochemical and clinical hyperandrogenism, IR, IGT, hyperglycemia, hyperlipidemia, infertility, and adverse obstetric outcomes (7). Among infertile PCOS women seeking assisted reproductive treatment (ART), obesity and dyslipidemia have been reported to be associated with *in vitro* fertilization/intracytoplasmic sperm injection (IVF/ICSI) outcomes. A BMI beyond the normal range has been reported to have adverse effects on ovarian response (10), retrieved oocyte count (10, 11), oocyte maturation (11), clinical pregnancy (12), and live birth (13, 14). BMI is also reported to be a risk factor for miscarriage in PCOS women undergoing IVF/ICSI (10, 13). Increased serum total cholesterol (TC) level has been found to decrease the chance of live birth in PCOS women undergoing IVF/ICSI (14, 15). The mechanism of obesity-related adverse ART outcomes of PCOS may be attributed to the inflammatory response, oxidative stress, and epigenetic alterations (16–19). However, few studies have directly analyzed the potential intermediate mechanisms by which obesity and dyslipidemia affect IVF/ICSI outcomes in PCOS women.

PCOS is considered to be a chronic inflammatory disorder associated with obesity (5, 16). Studies have shown elevated levels of inflammatory markers in PCOS women, including white blood cell (WBC) count, C-reactive protein (CRP), complement element 3 (C3), tumor necrosis factor-α (TNF-α), interleukin 6 (IL-6), interleukin 18 (IL-18), monocyte chemoattractant protein-1 (MCP-1), and macrophage inflammatory protein-1α (MIP-1α) (19, 20). In PCOS women, obesity, pro-inflammatory response, oxidative stress, IR, hyperinsulinemia, and hyperandrogenism interact to form a vicious cycle (7, 19). Inflammation has been elucidated to cause damage to oocyte and follicular quality (21). A uterine hyperinflammatory state of PCOS women may contribute to placental insufficiency and obstetric complications (22). Therefore, we assume the potential mediating role of inflammation on the association between adiposity and lipid metabolism indicators and IVF/ICSI outcomes in PCOS women.

In this study, we aimed to conduct mediation analyses to determine whether WBC count, the representative inflammatory marker, mediates the effect of adiposity and lipid metabolism indicators on IVF/ICSI early reproductive outcomes and pregnancy outcomes in PCOS women. This study follows the principles of the AGReMA guidelines (23).

## Materials and methods

### Ethical statement

Approval for this study was obtained from the institutional review board of Peking University Third Hospital (No. 2021SZ– 011). Each woman in this study signed an informed consent.

### Study population

A total of 1,534 PCOS women aged between 20 and 45 years old who underwent their first fresh IVF/ICSI cycles with autologous oocytes at the Reproductive Center of Peking University Third Hospital setting between January 2018 and December 2020 were screened for eligibility. PCOS was diagnosed according to the 2003 Rotterdam criteria (2). All the women had at least two of the following three criteria and excluded other causes of hyperandrogenism and ovulation dysfunction (1): biochemical hyperandrogenism and/or hirsutism (2); oligo- or amenorrhea; (3) polycystic ovarian morphology on transvaginal ultrasound (≥ 12 antral follicles in unilateral or bilateral ovaries or ovarian volume > 10 cm^3^). The exclusion criteria were as follows: (1) history of iatrogenic ovarian injury; (2) uterine abnormality; (3) history of adrenal diseases, thyroid disorders, T2DM, hyperprolactinemia, and Cushing’s syndrome; (4) history of autoimmune disease and recurrent spontaneous abortion; (5) chromosomal abnormalities in either of the spouses; (6) receiving *in vitro* maturation (IVM) or pre-implantation genetic testing (PGT). We extracted the data on baseline characteristics and IVF/ICSI laboratory outcomes from the internal ART database derived from the electronic medical records. Well-trained follow-up staff collected the pregnancy outcomes by telephone interviews, including biochemical pregnancy, clinical pregnancy, and live birth.

### IVF procedures

The ovarian stimulation, ovulatory trigger, oocyte retrieval, insemination, embryo culture, and embryo transfer were performed following standardized protocols at our institution as previously described (24, 25). The ovarian stimulation regimen and initial gonadotropin dose were decided according to each woman’s BMI and ovarian reserve condition. The embryos were evaluated for fertilization on Day 1 and morphologically graded on Day 3 and Day 5. Up to two Day 3 embryos or Day 5 to 6 blastocysts were transferred by an experienced reproductive medicine specialist (25, 26).

### Laboratory assessment

Serum total triglyceride (TG), TC, high-density lipoprotein cholesterol (HDL-C), and low-density lipoprotein cholesterol (LDL-C) were measured *via* an automatic biochemical analyzer (7170A, HITACHI, Japan) within 3 months before ovarian stimulation. Peripheral blood WBC count was determined *via* a fully automatic blood cell analyzer (Sysmex XN, Japan) within 1 week before ovarian stimulation.

### Study outcomes

The study outcomes include serum peak estradiol (E2) level, serum luteinizing hormone (LH) level on the ovulatory triggering day, serum progestin (P) level on the ovulatory triggering day, retrieved oocyte count, metaphase II (MII) oocyte count, normally fertilized zygote count, normally cleaved embryo count, high-quality Day 3 embryo count, blastocyst formation count, biochemical pregnancy, clinical pregnancy, and live birth. An MII oocyte is a mature oocyte that converts into haploid gametes with the first polar body extruded. Normal fertilization involves two pronuclei (2PN) in the zygote. A high-quality Day 3 embryo is a five- to eight-cell embryo with less than 30% fragmentation and uniform cell size. Biochemical pregnancy is defined as positive serum β-hCG tested 2 weeks after embryo transfer. Clinical pregnancy is defined as the presence of a gestational sac with fetal heartbeats detected on the transvaginal ultrasound 3 to 4 weeks after embryo transfer. Live birth is defined as the delivery of a living fetus beyond 28 weeks of gestation. Multiple births are considered as one live birth. All pregnancy outcomes (i.e., biochemical pregnancy, clinical pregnancy, and live birth) were those after fresh-cycle embryo transfer.

### Statistical analysis

All the data analyses were performed *via* “R” software (version 4.0.3). We summarized participants’ baseline reproductive and cycle characteristics and presented them using median [interquartile range (IQR)] or *n* (%). Differences in baseline reproductive and cycle characteristics across the four BMI groups (underweight: <18.5 kg/m^2^, normal weight: 18.5– 25 kg/m^2^, overweight: 25 –30 kg/m^2^, and obesity: ≥30 kg/m^2^) were evaluated using Kruskal–Wallis tests for continuous variables and Chi-squared tests for categorical variables (or Fisher’s exact test where appropriate). BMI, TG, TC, HDL-C, LDL-C, and WBC count were ln-transformed for tests that required a normal distribution due to right skewness.

Multivariate generalized linear models were constructed to assess the associations between adiposity and lipid metabolism indicators and IVF/ICSI outcomes, the associations between adiposity and lipid metabolism indicators and WBC count, and the effect of WBC count on IVF/ICSI outcomes in PCOS women to evaluate whether further mediation analyses were indicated. A normal distribution and identity link function were specified for serum peak E2, LH, and P levels on the ovulatory triggering day; a Poisson distribution and log link function were specified for retrieved oocyte count, MII oocyte count, normally fertilized zygote count, normally cleaved embryo count, high-quality Day 3 embryo count, and blastocyst formation count; and a binomial distribution and logit link function were specified for biochemical pregnancy, clinical pregnancy, and live birth. We examined the following covariates as potential confounders based on previous knowledge of biological relevance and statistical considerations: age (continuous), AFC (continuous), basal FSH (continuous), duration of infertility (continuous), infertility type (primary vs. secondary infertility), ovarian stimulation regimen (GnRH antagonist protocol, long GnRHa protocol, and others), insemination technique (IVF vs. ICSI), transferred embryos number (one vs. two), and the timing of embryo transfer (Day 3, Day 5, and Day 6). According to the Change-in-Estimate Method, these covariates remained in the final multivariate models if they caused an over 10% change in the effect estimates for the abovementioned associations (27).

Mediation analyses were performed to estimate the total, direct, and indirect effects *via* the R mediation package to determine whether WBC count is a potential mediator. When the hypothesis of the mediation analyses holds, the direct effect indicates the effect of adiposity and lipid metabolism indicators on IVF/ICSI outcomes in PCOS women after controlling for WBC count, and the indirect effect is the estimated effect of adiposity and lipid metabolism indicators mediated *via* WBC count. The proportion of mediation by WBC count was calculated as the ratio of the indirect effect to the total effect. Two-sided *p* < 0.05 were considered statistically significant.

## Results

### Baseline reproductive and cycle characteristics

This study included 1,534 PCOS women who had undergone their first cycles of IVF/ICSI. For comparing the baseline reproductive and cycle characteristics, these women were stratified into four groups according to the BMI classification of the World Health Organization: underweight (<18.5 kg/m^2^, *n* = 51), normal weight (18.5– 25 kg/m^2^, *n* = 656), overweight (25– 30 kg/m^2^, *n* = 489), and obesity (≥30 kg/m^2^, *n* = 338) (**Table 1**). Their age tended to increase with the increase of BMI class (all *p* = 0.04). Women with higher BMI tended to have higher AFC and lower basal FSH (*p* < 0.001). With BMI class increased, serum TG, TC, and LDL-C increased, and serum HDL-C decreased (all *p* < 0.05). There was no significant difference in AMH, infertility type, ovarian stimulation regimen, insemination technique, and timing of embryo transfer across the four BMI groups.

**Table 1:** Baseline reproductive and cycle characteristics of the PCOS women undergoing the first fresh IVF/ICSI cycles.

Characteristics	BMI, kg/m^2^				*p* ^a^
	<18.5	18.5–25	25–30	≥30	
*n* = 1,534	*n* = 51	*n* = 656	*n* = 489	*n* = 338	
Age, years	30 (28, 33)	31 (29, 34)	31 (28, 34)	31 (28, 33)	0.04
AFC, *n*	13 (9, 19)	15 (9, 20)	15 (11, 20)	16 (12, 22)	<0.001
Basal FSH, mIU/ml	6.2 (5.3, 7.3)	6.4 (5.1, 7.8)	5.9 (4.7, 7.2)	5.7 (4.5, 7.0)	<0.001
AMH, ng/ml	5.6 (2.6, 8.4)	5.5 (2.7, 8.5)	4.9 (3.1, 7.5)	4.4 (2.9, 7.2)	0.14
TG, mmol/L	0.8 (0.6, 1.0)	1.0 (0.7, 1.5)	1.5 (1.0, 2.0)	1.5 (1.2, 2.3)	<0.001
TC, mmol/L	4.3 (4.0, 4.7)	4.5 (4.0, 5.0)	4.5 (4.0, 5.1)	4.6 (4.1, 5.1)	0.01
HDL-C, mmol/L	1.5 (1.3, 1.7)	1.3 (1.1, 1.5)	1.2 (1.0, 1.3)	1.1 (1.0, 1.3)	<0.001
LDL-C, mmol/L	2.5 (2.0, 2.8)	2.8 (2.4, 3.2)	2.9 (2.5, 3.5)	3.0 (2.6, 3.5)	<0.001
WBC count, ×10^9^/L	6.6 (4.8, 8.1)	6.2 (5.1, 7.5)	7.0 (5.9, 8.4)	7.5 (6.3, 8.9)	<0.001
Duration of infertility, years	2 (1, 4)	3 (1, 4)	3 (2, 5)	4 (2, 6)	<0.001
Infertility type					0.67
Primary	38 (74.5%)	443 (67.5%)	327 (66.9%)	234 (69.2%)	
Secondary	13 (25.5%)	213 (32.5%)	162 (33.1%)	104 (30.8%)	
Ovarian stimulation regimen					0.05
GnRH antagonist	40 (78.4%)	521 (79.4%)	397 (81.2%)	269 (79.6%)	
Long GnRHa	7 (13.7%)	116 (17.7%)	87 (17.8%)	63 (18.6%)	
Others^b^	4 (7.8%)	19 (2.9%)	5 (1.0%)	6 (1.8%)	
Insemination technique					0.14
IVF	30 (58.8%)	476 (72.6%)	354 (72.4%)	252 (74.6%)	
ICSI	21 (41.2%)	180 (27.4%)	135 (27.6%)	86 (25.4%)	
Transferred embryos number					0.03
1	0 (0.0%)	4 (0.6%)	0 (0.0%)	2 (0.6%)	
2	44 (86.3%)	555 (84.6%)	438 (89.6%)	277 (82.0%)	
Timing of embryo transfer					0.07
Day 3	49 (96.1%)	620 (94.5%)	476 (97.3%)	314 (92.9%)	
Day 5	1 (2.0%)	24 (3.7%)	7 (1.4%)	13 (3.8%)	
Day 6	1 (2.0%)	12 (1.8%)	6 (1.2%)	11 (3.3%)	

Numbers are median ± IQR (range), except for percentages.

PCOS, polycystic ovary syndrome; IVF, in vitro fertilization; ICSI, intracytoplasmic sperm injection; BMI, body mass index; AFC, antral follicle count; FSH, follicle-stimulating hormone; AMH, anti-Mullerian hormone; TG, triglyceride; TC, total cholesterol; HDL-C, high-density lipoprotein cholesterol; LDL-C, low-density lipoprotein cholesterol; WBC, white blood cell; GnRH, gonadotropin-releasing hormone; GnRHa, gonadotropin-releasing hormone agonist; IQR, interquartile range.

a p-values comparing the differences across the four BMI groups.

b Other protocols include the short GnRHa protocol, ultrashort GnRH antagonist protocol, and minimal stimulation protocol.

### Adiposity and lipid metabolism indicators and IVF/ICSI outcomes in PCOS women

All the multivariable models were adjusted for age, AFC, and infertility type for consistency (**Tables 2**, **3**). **Table 2** displays the effects of adiposity and lipid metabolism indicators on hormone levels on the ovulatory triggering day, oocyte development outcomes, and fertilization in PCOS women. We found that BMI and serum TG were negatively associated with peak E2 level, retrieved oocyte count, MII oocyte count, and normally fertilized zygote count (all *p* < 0.001) and positively associated with serum P level on the ovulatory triggering day (all *p* < 0.05). Serum HDL-C was positively associated with peak E2 level and normally fertilized zygote count (all *p* < 0.01). **Table 3** displays the effects of adiposity and lipid metabolism indicators on early embryo development outcomes and pregnancy outcomes in PCOS women. We found that increasing BMI and serum TG levels were associated with decreasing normally cleaved embryo count, high-quality Day 3 embryo count, and blastocyst formation count and caused lower chances of clinical pregnancy and live birth (all *p* < 0.05). We also found that serum HDL-C was positively associated with normally cleaved embryo count, high-quality Day 3 embryo count, and blastocyst formation count (all *p* < 0.01). Moreover, increasing serum LDL-C level was associated with decreasing normally cleaved embryo count and high-quality Day 3 embryo count (all *p* < 0.05).

**Table 2:** β/RR (95% CI) in hormone levels on the ovulatory triggering day, oocyte development outcomes, and fertilization associated with adiposity and lipid metabolism indicators among PCOS women undergoing their first IVF/ICSI cycles based on generalized linear models (*n* = 1,534)^a^.

Adiposity and lipid metabolism indicators^b^	Peak E2 levels^b^, pmol/L		LH levels^b^, mIU/ml		*p* levels^b^, nmol/L		Retrieved oocytes, *n*		MII oocytes, *n*		Normal fertilization, *n*	
	β (95% CI)	*p*	β (95% CI)	*p*	β (95% CI)	*p*	RR (95% CI)	*p*	RR (95% CI)	*p*	RR (95% CI)	*p*
BMI, kg/m^2^	−0.56 (−0.75, −0.38)	<0.001	0.10 (−0.20, 0.40)	0.52	0.18 (0.03, 0.33)	0.02	0.82 (0.74, 0.90)	<0.001	0.84 (0.76, 0.93)	<0.001	0.78 (0.69, 0.89)	<0.001
TG, mmol/L	−0.12 (−0.18, −0.07)	<0.001	−0.06 (−0.15, 0.03)	0.18	0.07 (0.02, 0.11)	<0.01	0.95 (0.92, 0.98)	<0.001	0.95 (0.92, 0.98)	<0.001	0.92 (0.89, 0.96)	<0.001
TC, mmol/L	−0.04 (−0.22, 0.13)	0.61	0.15 (−0.13, 0.43)	0.28	0.05 (−0.08, 0.19)	0.46	0.95 (0.87, 1.04)	0.26	0.95 (0.87, 1.05)	0.31	0.95 (0.85, 1.06)	0.32
HDL-C, mmol/L	0.21 (0.07, 0.35)	<0.01	−0.08 (−0.31, 0.16)	0.53	−0.07 (−0.18, 0.04)	0.23	1.06 (0.98, 1.14)	0.16	1.07 (0.99, 1.15)	0.09	1.18 (1.07, 1.29)	<0.001
LDL-C, mmol/L	−0.06 (−0.17, 0.06)	0.36	0.18 (−0.02, 0.37)	0.07	0.05 (−0.04, 0.14)	0.28	0.97 (0.91, 1.03)	0.25	0.97 (0.91, 1.03)	0.26	0.94 (0.87, 1.02)	0.13

a Adjusted for age (continuous), AFC (continuous), and infertility type.

b Transformed by natural logarithm.

**Table 3:** RR/OR (95% CI) in early embryo development outcomes and pregnancy outcomes associated with adiposity and lipid metabolism indicators among PCOS women undergoing their first IVF/ICSI cycles based on generalized linear models (*n* = 1,534)^a^.

Adiposity and lipid metabolism indicators^b^	Normal cleavage, *n*		High-quality Day 3 embryos, *n*		Blastocyst formation, *n*		Biochemical pregnancy		Clinical pregnancy		Live birth	
	RR (95% CI)	*p*	RR (95% CI)	*p*	RR (95% CI)	*p*	OR (95% CI)	*p*	OR (95% CI)	*p*	OR (95% CI)	*p*
BMI, kg/m^2^	0.79 (0.70, 0.89)	<0.001	0.79 (0.68, 0.91)	<0.01	0.64 (0.49, 0.84)	<0.01	1.84 (0.99, 3.43)	0.05	2.11 (1.13, 3.98)	0.02	3.00 (1.54, 5.88)	<0.01
TG, mmol/L	0.92 (0.89, 0.96)	<0.001	0.95 (0.91, 0.99)	0.01	0.85 (0.79, 0.92)	<0.001	1.13 (0.94, 1.36)	0.18	1.33 (1.10, 1.60)	<0.01	1.24 (1.02, 1.52)	0.03
TC, mmol/L	0.92 (0.82, 1.03)	0.14	0.91 (0.80, 1.04)	0.17	0.92 (0.72, 1.17)	0.50	1.15 (0.65, 2.03)	0.62	1.24 (0.70, 2.21)	0.46	1.25 (0.69, 2.30)	0.46
HDL-C, mmol/L	1.17 (1.07, 1.29)	<0.001	1.17 (1.05, 1.30)	<0.01	1.45 (1.18, 1.78)	<0.001	0.79 (0.49, 1.27)	0.33	0.75 (0.46, 1.21)	0.23	0.65 (0.39, 1.07)	0.09
LDL-C, mmol/L	0.92 (0.86, 1.00)	0.04	0.90 (0.82, 0.98)	0.02	0.88 (0.74, 1.04)	0.14	1.23 (0.83, 1.82)	0.30	1.24 (0.84, 1.85)	0.28	1.38 (0.91, 2.10)	0.13

a Adjusted for age (continuous), AFC (continuous), and infertility type.

b Transformed by natural logarithm.

### Adiposity and lipid metabolism indicators and WBC count in PCOS women

The associations between adiposity and lipid metabolism indicators and WBC count in PCOS women are shown in **Table 4**. After controlling for age, AFC, and infertility type, BMI, serum TG, serum TC, and serum LDL-C were positively associated with WBC count (all *p* < 0.001). Serum HDL-C was negatively associated with WBC count (all *p* < 0.001).

**Table 4:** β (95% CI) in WBC count associated with adiposity and lipid metabolism indicators among PCOS women undergoing their first IVF/ICSI cycles based on generalized linear models (*n* = 1,534)^a,b^.

Adiposity and lipid metabolism indicators	WBC count, ×10^9^/L	
	β (95% CI)	*p*
BMI, kg/m^2^	0.44 (0.35, 0.52)	<0.001
TG, mmol/L	0.13 (0.11, 0.15)	<0.001
TC, mmol/L	0.14 (0.06, 0.22)	<0.001
HDL-C, mmol/L	−0.25 (−0.31, −0.19)	<0.001
LDL-C, mmol/L	0.10 (0.04, 0.15)	<0.001

a Adjusted for age (continuous), AFC (continuous), and infertility type.

b Adiposity and lipid metabolism indicators and WBC count were transformed by natural logarithm.

### WBC count and IVF/ICSI outcomes in PCOS women

The associations between WBC count and IVF/ICSI outcomes in PCOS women are presented in **Table 5**. After controlling for age, AFC, and infertility type, increasing WBC count was significantly associated with decreasing MII oocyte count, normally fertilized zygote count, normally cleaved embryo count, and high-quality Day 3 embryo count (all *p* < 0.05).

**Table 5:** β/RR/OR (95% CI) in early reproductive and pregnancy outcomes associated with WBC count among PCOS women undergoing their first IVF/ICSI cycles based on generalized linear models (*n* = 1,534)^a^.

Early reproductive and pregnancy outcomes	WBC count^b^, ×10^9^/L	
	β/RR/OR (95% CI)	*p*
Peak E2 levels^b^, pmol/L	−0.07 (−0.18, 0.04)	0.21
LH levels^b^, mIU/ml	−0.05 (−0.05, 0.13)	0.60
P levels^b^, nmol/L	0.05 (−0.03, 0.14)	0.23
Retrieved oocytes, *n*	0.96 (0.90, 1.01)	0.13
MII oocytes, *n*	0.93 (0.88, 0.99)	0.02
Normal fertilization, *n*	0.91 (0.85, 0.98)	0.01
Normal cleavage, *n*	0.91 (0.85, 0.98)	0.01
High-quality Day 3 embryos, *n*	0.89 (0.82, 0.97)	<0.01
Blastocyst formation, *n*	0.86 (0.73, 1.01)	0.07
Biochemical pregnancy	1.30 (0.90, 1.87)	0.16
Clinical pregnancy	1.25 (0.86, 1.81)	0.24
Live birth	1.38 (0.93, 2.04)	0.11

a Adjusted for age (continuous), AFC (continuous), and infertility type.

b Transformed by natural logarithm.

### Mediation analyses

**Figure 1:**
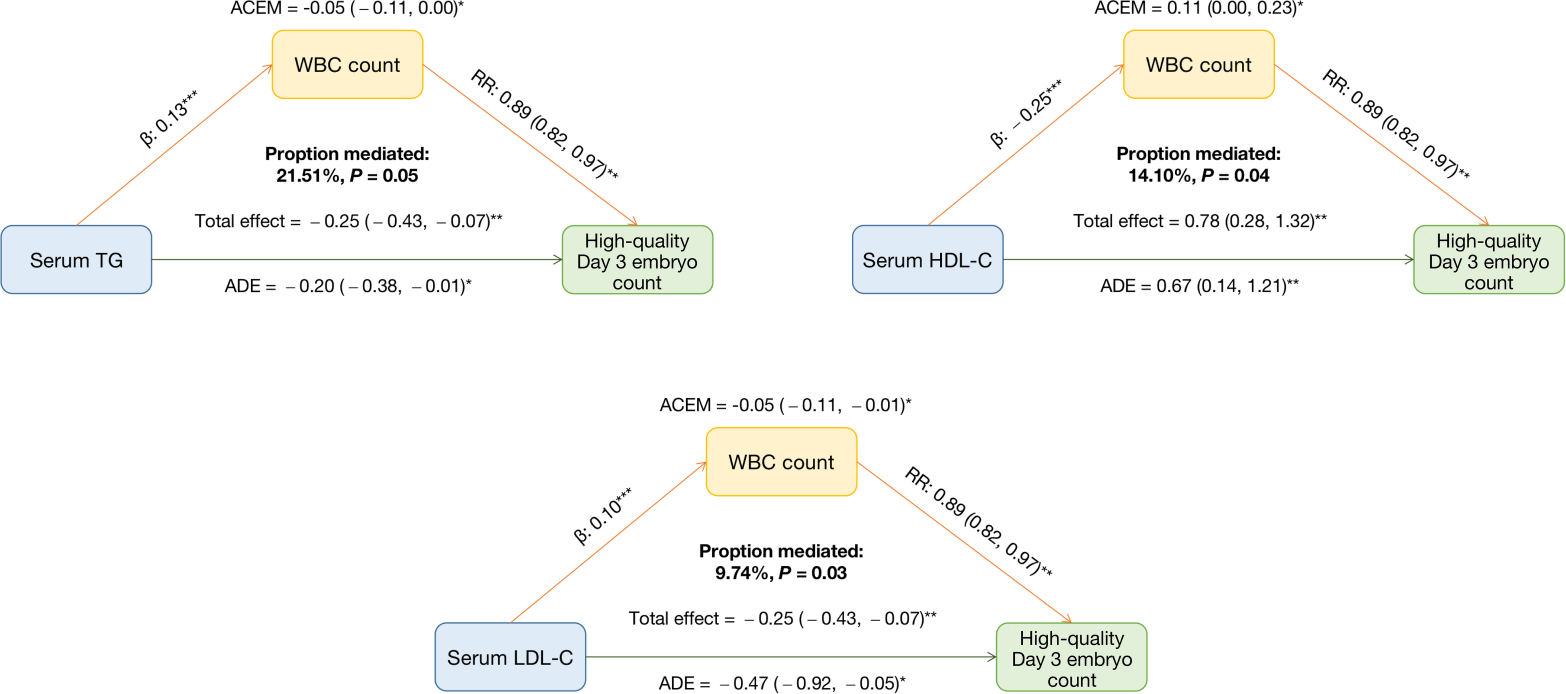
Mediating effects of WBC count on the associations between adiposity and lipid metabolism indicators and high-quality Day 3 embryo count. Adjusted for age (continuous), AFC (continuous), and infertility type. Adiposity and lipid metabolism indicators and WBC count were transformed by natural logarithm. ACME: average causal mediating effect. This represents the indirect effect, also known as the mediating effect of WBC count on the associations between adiposity and lipid metabolism indicators and high-quality embryo count. ADE: average direct effect. This represents the effects of adiposity and lipid metabolism indicators on high-quality Day 3 embryo count when WBC count is controlled. *p < 0.05. **p < 0.01.

Given that WBC count was associated with both adiposity and lipid metabolism indicators and IVF/ICSI outcomes, we assumed that WBC count could be a possible mediator. **Table 6** and **Figure 1** show the result of mediation analyses. After controlling for confounders and WBC count, we found significant adverse effects of serum TG and LDL-C on high-quality Day 3 embryo count (all *p* < 0.01). A significant mediating effect by WBC count was observed for the impact of serum TG and LDL-C on high-quality Day 3 embryo count, with 21.51% and 9.75% proportion mediated, respectively (all *p* < 0.05). We also found significant positive associations between serum HDL-C and high-quality Day 3 embryo count (*p* < 0.01). A significant mediating effect by WBC count was observed for the association between serum HDL-C and high-quality Day 3 embryo count, with 14.10% proportion mediated (*p* < 0.05).

**Table 6:** Mediation analyses investigating whether WBC count mediated the associations between adiposity and lipid metabolism indicators and high-quality Day 3 embryo count ^a,b^.

Mediators	Associations	Total effect (95% CI)	Mediated effect (95% CI)	Estimated proportion mediated (%)
WBC count	High-quality Day 3 embryo count and TG	−0.25 (−0.43, −0.07)**	−0.05 (−0.11, 0.00)*	21.51
	High-quality Day 3 embryo count and HDL-C	0.78 (0.28, 1.32)**	0.11 (0.00, 0.23)*	14.10
	High-quality Day 3 embryo count and LDL-C	−0.52 (−0.98, −0.10)**	−0.05 (−0.11, −0.01)*	9.75

a Adjusted for age (continuous), AFC (continuous), and infertility type.

b Adiposity and lipid metabolism indicators and WBC count were transformed by natural logarithm.

*p < 0.05.

**p < 0.01.

## Discussion

In this retrospective study among PCOS women, we found significant dose-dependent associations between adiposity and lipid metabolism indicators and IVF/ICSI outcomes, including hormone levels on the ovulatory triggering day, oocyte development outcomes, fertilization, early embryo development outcomes, and pregnancy outcomes. We also found significant dose-dependent associations between adiposity and lipid metabolism indicators and WBC count, as well as between WBC count and IVF/ICSI outcomes. Further analyses elucidated that WBC count partially mediates the adverse effects of serum TG and LDL-C on high-quality Day 3 embryo count in PCOS women. We also found that WBC count partially mediates the association between serum HDL-C and high-quality Day 3 embryo count in PCOS women.

PCOS and obesity have an intense and complex association (28). Evidence from Mendelian randomization (MR) study suggested that an increase in body fat distribution indicators (i.e., BMI and waist-to-hip ratio) is causally related to PCOS (29). Obesity increases hyperinsulinemia and IR, subsequently increasing ovarian androgen production (30, 31). The excessive adipose tissue can aromatize these androgens to estrogens, which are then released into the circulation and interfere with the function of the hypothalamic–pituitary–ovarian (HPO) axis (28). Moreover, obesity increases inflammatory adipokines, which forms a vicious feedback cycle with hyperinsulinemia (7). These alterations exert a significant impact on PCOS-related symptoms. Obesity and dyslipidemia worsen all PCOS’s clinical manifestations and reproductive outcomes, including menstrual disorder, biochemical and clinical hyperandrogenism, hyperglycemia, metabolic syndrome, infertility, miscarriage, gestational diabetes mellitus, and pregnancy-induced hypertension (7, 32–34). Obesity and dyslipidemia may also damage oocyte and embryo quality. Several pieces of evidence can support the negative impact of obesity on ART outcomes in PCOS women. Among PCOS women undergoing ART, obesity can lead to poor ovarian response and disturb oocyte maturation (10, 11). Adiposity and lipid metabolism indicators have also been frequently reported as risk factors for clinical pregnancy and live birth in PCOS women (12–15). In this study, we observed that adiposity and lipid metabolism indicators are negatively associated with PCOS women’s IVF/ICSI outcomes, including ovarian response indicators, oogenesis outcomes, embryogenesis outcomes, and pregnancy outcomes. These results provide complementary evidence for the above-related studies.

Obesity and dyslipidemia are involved in the low-grade chronic inflammatory progress in PCOS women (5, 16, 19). The hematologic inflammatory markers in PCOS women have been reported to be closely related to obesity (19, 35, 36). Consistently, we found significant correlations between the PCOS women’s lipid metabolism indicators and WBC count, a representative inflammatory marker. Studies have elucidated that excessive adipose tissue can cause the imbalance of the immune system and activate pro-inflammatory processes, characterized by high levels of TNF-α, pro-inflammatory interleukins, and chemokines (3, 37–39). The mechanisms triggering the inflammatory state in obese women have also been extensively studied, including cell enlargement and death in the obese tissue, imbalanced adipokine production, fatty acid dyshomeostasis, local hypoxia, mitochondria dysfunction, dysbiosis in the gut microbiome, mechanical stress, and endoplasmic reticulum stress (3, 19, 40). Moreover, epigenetic regulations, including DNA methylation, histone acetylation, and changes in noncoding RNA levels, have been reported to affect the inflammatory response and oxidative stress in PCOS women (16).

Obesity-induced excessive pro-inflammatory mediators may contribute to the occurrence and progress of PCOS by various mechanisms. Pro-inflammatory mediators may inhibit insulin receptors, reduce insulin sensitivity and aggravate IR, consequently worsening reproductive dysfunction (41, 42). Obesity can induce higher levels of inflammatory mediators in the ovary, which may cause irreversible damage to the oogenesis and ovary function (21, 43). Oxidative stress, closely related to inflammatory response, can also lead to oocyte apoptosis and ovarian dysfunction (44). The change in obesity-induced inflammation and oxidative stress pathways may alter the expression of genes related to oocyte quality, thus adversely affecting the subsequent embryogenesis (21). Furthermore, the NLRP3 inflammasome and the chemokine decoy receptor D6, two representative uterine inflammatory mediators, may disturb the maternal–fetal interface with the subsequent occurrence and progress of obstetric complications (22). Existing clinical studies have elucidated the adverse effects of inflammatory mediators on female reproductive outcomes. According to a meta-analysis of 22 studies, peripheral NK cells are associated with recurrent miscarriage (45). Increasing circulating CRP in the pre-implantation period is related to adverse ART outcomes, including folliculogenesis disorder, poor oocyte competence, abnormal embryo development, and poor endometrial receptivity (46). Moreover, hematologic inflammatory markers (i.e., WBC count and neutrophils) have been reported to be negatively associated with oocyte developmental competence in PCOS women (35). Mean platelet volume (MPV) has been reported to correlate significantly to the clinical pregnancy rate among PCOS women undergoing ART (35).

This study found that WBC count was a significant mediator for the associations between adiposity and lipid metabolism indicators (i.e., serum TG, HDL-C, and LDL-C) and high-quality Day 3 embryo count, suggesting that inflammation may mediate the adverse effect of obesity and dyslipidemia on embryogenesis. As we have discussed above, obesity and dyslipidemia have been illuminated to be associated with hematologic inflammatory changes in PCOS women and are the potential causality of PCOS’s low-grade chronic inflammatory state (19). Studies also supported the adverse effect of obesity-induced excessive pro-inflammatory mediators on oogenesis and embryogenesis (21). However, the results of these mediation analyses need to be interpreted with caution since solid assumptions are required for the causal interpretation of the findings.

Although cautious interpretation is needed for the results of our mediation analysis, our findings explained the potential inflammatory response mechanism of obesity-induced embryo development impairment in PCOS women and provided potential therapeutic targets. Anti-inflammatory therapy was found to help alleviate PCOS. In a PCOS mouse model, as a kind of stem cell therapy, transplantation of bone marrow mesenchymal stromal cells (BM-MSCs) reduced serum malondialdehyde (MDA), TNF-α, and IL-6 concentrations and improved folliculogenesis (47). Another animal study found that inulin and metformin improved ovarian morphology and endocrine function *via* anti-inflammation and modulating gut microbiota (48). Saffron petal extract and saffron petal anthocyanins have been reported to ameliorate symptoms of PCOS *via* improving dysregulation of ovarian steroidogenesis, antioxidant enzymes, and inflammatory response (49). Resveratrol has been demonstrated to have anti-inflammatory effects in PCOS women by suppressing the expression of NF-κB and NF-κB-regulated gene products (50). There has been considerable progress in the research of antioxidants in anti-inflammation and improving IVF outcomes in PCOS women. In clomiphene-citrate-resistant PCOS women, the combined application of coenzyme Q10 and clomiphene citrate improves ovulation and clinical pregnancy rates (51). A recent randomized controlled trial elucidated that quercetin consumption reduced serum TNF-α and IL-6 concentrations and improved oocyte quality, embryo grade, and the pregnancy rate in PCOS women (52). There are also some emerging therapies under investigation. Adipokine-targeted regimens (i.e., recombinant protein, therapeutic peptide, monoclonal antibody, adipokine receptor agonist, and adipokine receptor antagonist) can improve obesity, pro-inflammatory response, IR, and ovarian dysfunction, thus may be a novel therapeutic strategy for PCOS (3). Moreover, as PCOS is a chronic inflammatory state associated with obesity, lifestyle interventions focused on diet, exercise, and behavioral or combined treatments, and anti-obesity medications still need to be promoted and encouraged in PCOS patients (7).

This is the first study focused on the mediating role of inflammation in the associations between PCOS women’s adiposity and lipid metabolism indicators and IVF/ICSI outcomes. However, some limitations in this study still warrant consideration. First, we conducted a retrospective study, thus possibly causing bias in data collection and making it difficult to determine causation. However, the objective nature and specific definitions of the indicators we studied may eliminate bias to some extent. Second, we did not consider adjunctive therapy (i.e., diet, exercise, or medication) before or during IVF/ICSI treatment. There are also some other adiposity and lipid metabolism indicators (i.e., waist circumference, hip circumference, waist-to-hip ratio, body fat percentage, skinfold thickness, lipoproteins, and apolipoproteins). Moreover, many other inflammatory markers have been proposed to be associated with PCOS (53). Future prospective studies can address these issues in a better experimental design. Third, serum lipid and peripheral blood WBC count testing was performed only once and may not be at the same time point. Moreover, the timing of laboratory assessment of some PCOS women may be long before ovarian stimulation. This affected the representativeness of the data. Future studies with more rigorous designs strictly specifying the timing of specimen collection and using repeated measures are still warranted. Fourth, we simultaneously performed multiple hypothesis tests to assess the correlations between adiposity and lipid metabolism indicators and WBC count. Therefore, the possibility that our findings were due to chance cannot be excluded entirely. Fifth, our study population was from a reproductive center, which limits the extrapolation of our results to the general PCOS population due to potential selection bias. However, these findings may have implications for PCOS women seeking infertility treatment, which accounts for 40% to 72% of PCOS women (1, 54).

In conclusion, we found that lipid metabolism dysfunction was associated with WBC count in PCOS women. We also found that WBC count was negatively associated with PCOS women’s IVF/ICSI outcomes (i.e., MII oocyte count, normally fertilized zygote count, normally cleaved embryo count, and high-quality Day 3 embryo count). As a representative inflammatory marker, WBC count partially mediated the association between adiposity and lipid metabolism indicators (i.e., serum TG, HDL-C, and LDL-C) and high-quality Day 3 embryo count. These findings show the importance of the inflammation-related mechanism on the obesity- and dyslipidemia-induced abnormal embryogenesis in PCOS women.

## Data availability statement

The original contributions presented in the study are included in the article/supplementary material. Further inquiries can be directed to the corresponding authors.

## Ethics statement

The studies involving human participants were reviewed and approved by institutional review board of Peking University Third Hospital. The patients/participants provided their written informed consent to participate in this study.

## Author contributions

JQ and XL conceived and designed the research. LC and HJ collected the clinical data. HJ analyzed the data and wrote the draft. TT and HS provided statistical advice. JQ, XL, HC, RY, and NH critically revised the paper. All the authors contributed to the article and approved the submitted version.
